# A Novel Variant in an Israeli Bedouin Family: The First Reported Cases of Carbonic Anhydrase VA (CA5A) Deficiency in Israel

**DOI:** 10.3390/genes17050537

**Published:** 2026-05-01

**Authors:** Nitzan Abelson, Eyal Kristal, Eli Hershkovitz, Ohad Wormser, Vadim Dolgin, Shirly Amar, Orna Staretz-Chacham

**Affiliations:** 1 Pediatrics Department A, The Cheryl and Haim Saban Children’s Hospital, Soroka University Medical Center, Ben-Gurion University, P.O. Box 151, Beer Sheva 84101, Israel; 2 Pediatric Day Hospitalization Unit, The Cheryl and Haim Saban Children’s Hospital, Soroka University Medical Center, Ben-Gurion University, P.O. Box 151, Beer Sheva 84101, Israel; 3 Pediatrics Department D, The Cheryl and Haim Saban Children’s Hospital, Soroka University Medical Center, Ben-Gurion University, P.O. Box 151, Beer Sheva 84101, Israel; 4 The Morris Kahn Laboratory of Human Genetics, Faculty of Health Sciences, Ben-Gurion University, P.O. Box 151, Beer Sheva 84101, Israel; 5 Genetics Institute, Soroka University Medical Center, P.O. Box 151, Beer Sheva 84101, Israel; 6 The Shraga Segal Department of Microbiology, Immunology and Genetics, Faculty of Health Sciences, Ben-Gurion University, P.O. Box 151, Beer Sheva 84101, Israel; 7 The Center for Rare Diseases, The Cheryl and Haim Saban Children’s Hospital, Soroka University Medical Center, Ben-Gurion University, P.O. Box 151, Beer Sheva 84101, Israel

**Keywords:** carbonic anhydrase VA (CA5A) deficiency, hyperammonemia, encephalopathy, lactic acidosis, ketonuria

## Abstract

Carbonic anhydrase VA (CA5A) deficiency (OMIM 615751) is an ultra-rare inborn error of metabolism, presenting in newborns, infants, and young children with a pentad of encephalopathy, hyperammonemia, lactic acidosis, ketonuria, and hypoglycemia. We present two cases: a case of a healthy Bedouin infant admitted with hyperammonemic encephalopathy that required urgent hemodialysis, and her younger sibling, who presented with a milder episode. Molecular analysis confirmed the diagnosis of CA5A deficiency due to a homozygous missense variant in the *CA5A* gene. Both patients had a favorable outcome with continued normal development. These were the first identified cases of CA5A deficiency in the Bedouin population, emphasizing the importance of a high index of suspicion, early genetic consultation and diagnosis, and prompt treatment at the earliest possible stage of a hyperammonemic crisis.

## 1. Introduction

Carbonic anhydrase VA (CA5A) deficiency (OMIM 615751) is an extremely rare inborn error of metabolism, first reported by van Karnebeek et al. in 2014 [1]. CA5A is a zinc metalloenzyme, located mainly in the liver, kidney, and skeletal muscles [2], which provides bicarbonate as a substrate to various enzymes in the mitochondria, including carbamoyl phosphate synthetase 1 (CPS1), pyruvate carboxylase (PC), propionyl-CoA carboxylase (PCC), and 3-methylcrotonyl-CoA carboxylase (3-MCC). As a result, CA5A deficiency impairs the urea cycle, tricarboxylic acid cycle, and gluconeogenesis [3,4] with corresponding clinical manifestations. The spectrum of onset varies between the first days of life and 2 years of age. Most children present with a characteristic pentad of encephalopathy (including lethargy, feeding difficulties, weight loss, tachypnea, seizures, and coma), hyperammonemia, lactic acidosis, ketonuria, and hypoglycemia [5,6,7].

The disease is inherited in an autosomal recessive pattern. Fewer than 50 affected individuals have been reported to date. Long-term outcomes are generally favorable, with most patients achieving normal psychomotor development and no metabolic crises [6,8,9]. Only a third of patients may present with recurrent metabolic crises triggered by catabolism [10].

## 2. Case Report

### 2.1. Case Presentation

Case 1: A previously healthy 9-month-old female infant, the third-born child to non-consanguineous parents of Bedouin origin, was admitted to the Emergency Department due to an intermittent fever accompanied by mild gastroenteritis, reduced intake, and upper respiratory symptoms for nine days prior to admission. On the day of admission, her parents noted an acute onset of increasing weakness and apathy, a marked change compared with the preceding days. She was born at term following an uneventful pregnancy and underwent normal newborn screening. Anthropometric indices were appropriate for age.

Upon admission, she was encephalopathic, lethargic, and unresponsive, and was reported to have a generalized seizure. Vital signs were normal except for tachycardia of 154/min. Physical examination revealed hepatomegaly, and a full neurological examination on admission demonstrated encephalopathy characterized by apathy and hyperreflexia.

Case 2: The younger brother of the patient in case 1, aged 14 months at the time of presentation, was admitted to the emergency room due to gastroenteritis, without apparent signs of severe dehydration or any neurological impairments. Upon admission, the parents informed the physician of his sister’s diagnosis and actively requested a serum ammonia level test. It is noted that he, like his sister, was born at term, following an uneventful pregnancy, and underwent normal newborn screening. He, too, had anthropometric indices appropriate for age.

### 2.2. Diagnostic Evaluation

Case 1: Laboratory tests demonstrated (Table 1) a normal glucose level (81 mg/dL) with an anion gap (AG) of 24 mEq/L and a compensated metabolic acidosis (pH 7.37, pCO_2_ 23 mmHg, HCO_3_ 13.3 mmol/L), serum lactate was 6.4 mmol/L (normal <2.1 mmol/L), and +4 urine ketones (by keto stick). Her blood count, coagulation tests, and electrolytes were within normal ranges. Serum chemistry demonstrated elevated alanine aminotransferase (370 U/L; normal 0–31 U/L) and highly elevated serum ammonia (689.8 µg/dL; normal <80 µg/dL). Brain imaging by CT was unremarkable, and a lumbar puncture demonstrated an elevated cerebrospinal fluid (CSF) lactate level of 5.4 mmol/L with no pleocytosis. Brain MRI was normal.

Urine organic acid analysis demonstrated marked excretion of lactate and ketones, with dicarboxylic aciduria and no specific diagnostic pattern. Plasma amino acid analysis demonstrated a normal citrulline level (15 µmol/L), a normal orotic acid level (1.8 µmol/L), and a low glutamine level (130 µmol/L).

As part of the work-up, and due to a high rate of under-reported consanguinity in the Bedouin population and a high suspicion of a urea cycle defect; Clinical Exome sequencing (performed by CeGaT with the TwistPlus capture kit, achieving an average coverage of ×177 and 99.36% of target regions covered above ×20) was performed, reporting only VUS (variant of uncertain significance), likely pathogenic, or pathogenic variant related to hyperammonaemia. Only a single variant was reported, *CA5A*: c.292A>G, p.Thr98Ala (NM_001739.2) variant, classified as a VUS, found in a homozygote state in the patient.

Case 2: Laboratory tests demonstrated normal blood counts, coagulation tests, and electrolytes. Blood chemistry showed elevated alanine aminotransferase (222 U/L; normal 0–31 U/L), and initial serum ammonia was 199 µg/dL on admission. In addition, a high anion gap metabolic acidosis was present upon admission.

**Figure 1:**
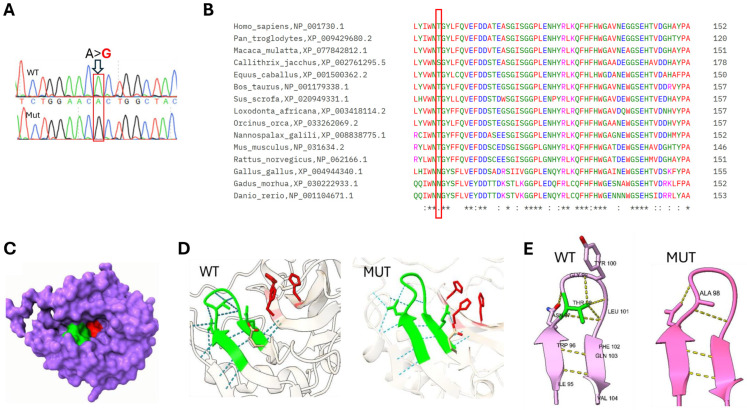
**Sanger sequencing, evolutionary conservation, and a three-dimensional model of CA5A depicting the predicted structural impact of the Thr98Ala variant on the catalytic site and zinc-binding coordination**. (**A**) Sanger sequencing chromatogram showing both the wild-type (unrelated individual) and mutant alleles (within case-patient n.2). (**B**) Multiple sequence alignment of selected CA5A orthologs using Clustal Omega (version 1.2.4). The affected amino acid Thr98 is highlighted in red (different colors are attributed based on the amino acid physicochemical properties, the symbol below each column running from ‘*’ to ‘:’ and ‘.’ or none- mark the level of conservation from highest to none, when ‘.’ means semi-conserved or replaced by one having similar characteristics). (**C**) Surface topography of the CA5A catalytic funnel and active site access window. Space-filling representation of the mature human mitochondrial CA5A protein, illustrating the deep architecture of the active site cavity. The three essential catalytic histidine residues (His130, His132, and His155; highlighted in red) are located at the base of the funnel, where they coordinate the catalytic zinc Ion. The ß-hairpin turn (residues 95–104; around Thr98- highlighted in green) is thought to form a rim guarding the entrance to the reaction chamber. (**D**) The catalytic Zn2+ is tetrahedrally coordinated by the three highly conserved histidine residues (shown in red). The T98A mutation (within the ß-hairpin turn, shown in green) is hypothesized to propagate structural shifts that distort this precise geometry, potentially impairing the enzyme’s affinity for zinc or its catalytic efficiency. (**E**) Predicted Structural destabilization of the beta-hairpin turn by the T98A mutation (hydrogen bonds are marked with dashed yellow lines). Threonine 98 is predicted to serve as a vital “structural anchor” situated at the apex of a ß-hairpin turn. This “anchor” is believed to be lost when the non-polar alanine is found in place.

Using targeted Sanger sequencing (primers F: 5′-tcactcagggtctccgtctc-3′; R: 5′-cctcaagcgagtctctgtcc-3′, directed to the sibling’s variant), *CA5A*: c.292A>G, p.Thr98Ala (NM_001739.2) was found in this patient as well (Figure 1).

The c.292A>G (p.Thr98Ala) variant in *CA5A* is exceptionally rare globally and is absent from the Genome Aggregation Database (gnomAD v4.1.1) and GME Variome (based on 2547 exomes from diverse Middle Eastern populations). Interestingly, in our internal genomic database and shared community, the variant was identified in a heterozygous state in 2 out of more than 2000 ethnically matched alleles (minor allele frequency < 0.1%). This global absence fulfills the criteria for PM2_Supporting. Note that a highly specific clinical and biochemical footprint is a hallmark of carbonic anhydrase VA deficiency, providing phenotypic support for pathogenicity (thereby, according to ACMG classification, PP4_Supporting).

The variant is highly conserved across all examined mammals (Figure 1). Protein Multiple Sequence Alignment was performed using the Clustal Omega program [11]. Note that Threonine is replaced in distantly related vertebrates by Serine or Asparagine, amino acids with similar polar uncharged side chains. In silico evaluation of the p. Thr98Ala variant yielded discordant results. While tools such as DANN, GENOCANYON, and fitCons indicated a deleterious effect, standard sequence-based meta-predictors such as REVEL and BayesDel did not predict pathogenicity. To maintain rigorous adherence to ACMG variant interpretation guidelines, we have opted not to apply the computational evidence criterion (PP3 not applied) because of the conflicting meta-predictor scores. However, sequence-only tools often underestimate the impact of mutations in structural loops, as they may fail to capture the 3D geometric requirements of specific hydrogen-bond donors required for distal active-site integrity [12,13,14]. Controversially, 3D structural modeling highlights the variant as highly suspicious. As detailed in our AlphaFold modeling (Figure 1), the T98A substitution apparently abolishes a critical hydrogen-bonding network. This loss is predicted to cause a localized structural collapse of the beta-hairpin and a subsequent distortion of the distal zinc-binding site. Although direct parental testing was unavailable to perform standard trio-based segregation analysis, the familial and demographic context provides substantial genetic evidence. The patients belong to the Bedouin population of the Negev, a community characterized by well-documented high rates of consanguinity and endogamy. Within this specific population architecture, the identification of an ultra-rare, homozygous variant co-segregating in two affected siblings strongly implies identity-by-descent from a common ancestral carrier, effectively confirming in-trans inheritance. While formal haplotype analysis is required to definitively prove a founder block, the presence of the exact homozygous variant in multiple affected family members within a highly endogamous framework justifies the cautious application of the segregation criterion at a supporting level (PM3_Supporting).

Based on the aggregation of three supporting criteria (PM2_Supporting, PM3_Supporting, PP4_Supporting), the variant is formally classified as a Variant of Uncertain Significance (VUS) according to the strict application of ACMG guidelines. However, given the specific clinical phenotype, the endogamous population context, and the compelling structural rationale for a severe loss-of-function defect, we consider this a highly suspicious “hot VUS.” This classification underscores the limitation of current in silico tools for ultra-rare structural variants and highlights the critical need for future in vitro enzymatic assays to definitively confirm pathogenicity.

### 2.3. Treatment

Case 1: Due to an initial clinical differential diagnosis of infectious meningitis or encephalitis, which may present initially with normal inflammatory markers and a metabolic crisis, she was treated immediately with IV ceftriaxone and acyclovir, accompanied by cessation of protein intake and initiation of intravenous fluid therapy with 10% glucose. Additionally, ammonia scavengers (sodium benzoate and sodium phenylbutyrate) were initiated together with arginine supplementation. The patient was transferred for hemodialysis and, within a single cycle, experienced a rapid decrease in ammonia levels to normal (under 24 h since the initial presentation) and clinical improvement. Laboratory tests at discharge are presented in Table 2.

Case 2: He was hospitalized and treated according to the current standard of care, with intravenous fluid therapy with 10% glucose, intravenous lipid emulsion, and ammonia scavengers—sodium benzoate and sodium phenylbutyrate—with arginine supplementation. The patient improved quickly with no need for hemodialysis. Throughout the hospitalization, no encephalopathy was recorded.

### 2.4. Outcome

Both siblings continue their follow-ups at the metabolic clinic with no recurrent events and follow the recommendation of a protein-restricted diet during infections. So far, their developmental milestones have been appropriate for age, after two years of follow-up.

## 3. Discussion

We present two cases of CA5A deficiency, a rare and underdiagnosed autosomal recessive metabolic disease first reported by van Karnebeek et al. in 2014 [1] and, to date, reported in fewer than 50 patients [15]. Several explanations may be found for the relatively benign clinical course observed in CA5A deficiency patients, one being the overlapping function of the CA5B enzyme, and the other being that bicarbonate can be partially produced via a non-enzymatic reaction [3]. Nonetheless, early suspicion and management can be lifesaving and improve prognosis in this rare disorder [2,5].

**Figure 2:**
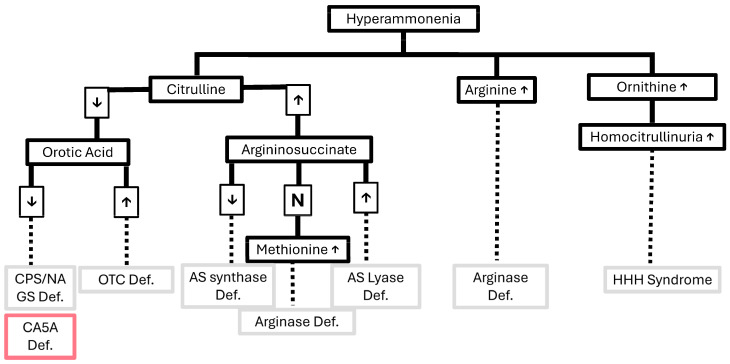
**A suggested diagnostic approach for the assessment of suspected urea cycle defects.** AS, argininosuccinic acid; CPS, carbamoyl phosphate synthetase I; Def., deficiency; HHH, Hyperornithinemia-Hyperammonemia-Homocitrullinuria; N, normal; OTC, ornithine transcarbamylase; NAGS, N-acetylglutamate synthase; ↑, high; ↓, low.

The first case presented with metabolic acidosis and a compensatory respiratory alkalosis with hyperammonemia, which could be suggestive of acute gastroenteritis triggering an underlying urea cycle disorder. The differentiation of urea cycle defects usually relies on supporting laboratory tests, such as acid–base status, metabolic profile, and plasma amino acids, as outlined in established urea cycle defect guidelines [6,16]. CA5A deficiency, while mimicking proximal urea cycle disorders, is associated with lactic acidosis and with elevated plasma glutamine and low plasma citrulline, which is likely due to secondary CPS1 deficiency, and is non-specific for CA5A [17]. A figure suggesting an algorithmic approach for a patient with a suspected urea cycle defect is presented in Figure 2 [18]. In the differential diagnosis of our case, a late-onset urea cycle disorder precipitated by an acute febrile illness was initially considered; however, the presence of metabolic acidosis, rather than the respiratory alkalosis typically seen in such disorders, was attributed to vomiting, diarrhea, and dehydration. In hyperammonemia due to urea cycle defects, glutamine levels are typically markedly elevated. In this case, the finding of low glutamine levels, together with normal citrulline and orotic acid and the absence of argininosuccinic acid, effectively argues against a urea cycle disorder as the underlying cause. Low glutamine in the presence of hyperammonemia may be seen in organic acidemias (e.g., propionic, isovaleric, or methylmalonic acidemia) with secondary inhibition of the urea cycle; however, there was no supporting evidence for this on the acylcarnitine profile from newborn screening. The presentation could also be compatible with severe pyruvate carboxylase deficiency, although this condition typically presents in the neonatal period and is associated with elevated citrulline levels. E3 deficiency was also considered; however, branched-chain amino acid levels and citrulline were normal on screening, and there was no hypoglycemia. A mitochondrial disorder, such as TMEM70 deficiency, could not be excluded, although the normal citrulline does not support this diagnosis. Overall, the biochemical pattern is most consistent with carbonic anhydrase VA deficiency. For these reasons, genetic testing (whole exome sequencing) was performed.

While no clinical diagnostic criteria for CA5A deficiency have been published so far [6], the diagnosis of CA5A deficiency is proposed in children with a clinical course of hyperammonemic encephalopathy, metabolic acidosis, elevated plasma glutamine and alanine, low-to-normal plasma citrulline, and a urine organic acid analysis demonstrating metabolites suggestive of multiple carboxylase deficiency. Genetic analyses demonstrate biallelic pathogenic variants in *CA5A*, with considerable allelic heterogeneity; more than 19 variants have been reported in the HGMD Professional database. This genetic diversity complicates diagnosis, as novel disease-causing variants continue to emerge.

The pathogenicity of the T98A substitution in *CA5A* is hypothesized to arise from the loss of critical stabilizing interactions within a key structural turn. Structural modeling suggests that Thr98 is predicted to serve as a “structural anchor” situated at the apex of a β-hairpin turn. As shown in Figure 1, the hydroxyl group of Thr98 is predicted to act as a central hydrogen-bonding hub, forming stabilizing bonds with the peptide backbone of neighboring residues Gly99 and Leu101. This likely constrains the loop in a rigid conformation, maintaining proper alignment of the adjacent β-strands (residues 95–96 and 102–104). In contrast, the T98A mutation replaces the polar threonine with a non-polar alanine, whose methyl side chain is incapable of forming hydrogen bonds. Structural models suggest that this interaction network is disrupted (Figure 1), leading to reduced stability of the β-turn.

This predicted structural collapse can have two profound consequences for the active site. First, the relaxation of the 95–104 loop threatens to collapse the rim inward, physically occluding the catalytic funnel. This occlusion would directly obstruct substrate diffusion and disrupt the internal solvent network required for proton shuttling. Second, the structural shift induced by this local collapse is likely to distort the underlying active-site geometry, perturbing the spatial orientation of the zinc-binding histidines (His130, His132, and His155) and impairing metal coordination (see Figure 1). Overall, we propose that this defect impairs mitochondrial bicarbonate production, thereby disrupting the urea cycle and contributing to hyperammonemic encephalopathy. Further biochemical studies are needed to confirm the functional impact of this structural alteration.

This novel variant, found in both patients, is highly suspected of causing a clinical phenotype similar to that previously reported in patients with CA5A deficiency. Clinically, the presentation of our patients is consistent with previously reported cases of CA5A deficiency, particularly with respect to early-onset hyperammonemic encephalopathy, lactic acidosis, and favorable outcomes following treatment. This study presents a previously unreported *CA5A* variant, raising the possibility of a population-specific variant. Although current data are insufficient to establish a founder effect, this observation highlights the need for further genetic studies in this population, where consanguinity rates are relatively high. The identification of this variant substantiates the role of early genetic testing of patients with hyperammonemic encephalopathy to identify this disease, in which, with appropriate management, more than 60% of affected individuals may achieve an excellent long-term prognosis and favorable outcome [6,9,10] while avoiding the consequences of missed underlying metabolic derangements. Moreover, early diagnosis and intervention in case 2 led to a shorter hospitalization and the need for less aggressive, less costly treatment than in case 1, underscoring the immediate benefit of these tests.

Treatment has focused mainly on protein restriction and intravenous dextrose and additional calories in the form of lipid infusions upon signs of inadequate oral intake or metabolic decompensation, whilst monitoring blood levels of ammonia, serum lactate, glucose, blood gases, electrolytes, and liver enzymes [9]. The course of the disease may be fulminant and fatal despite rapid initiation of adequate treatment, with ammonia and lactate normalization, especially in those presenting at an older age [9,18]. Due to the life-threatening presentation, it is important to be aware of asymptomatic individuals with biallelic *CA5A* pathogenic variants and be prepared with a sick-day management plan to prevent metabolic decompensation during illness. This is done by adjusting diet, medications, hydration, and monitoring. In CA5A deficiency, this includes avoiding prolonged fasts (more than 3–8 h, depending on age) and providing frequent high-carbohydrate containing drinks. Frequent vomiting may require prompt medical supervision. Importantly, between episodes, blood tests may be normal apart from mildly elevated blood lactate levels and the presence of ketonuria, increasing the likelihood of undiagnosed individuals in the general population [1,6].

In conclusion, in this case series of CA5A deficiency, we present a novel missense variant leading to CA5A deficiency in two siblings of Bedouin origin, reinforcing the importance of genetic testing in all children presenting with a hyperammonemic crisis and encephalopathy. This report expands the mutational spectrum of CA5A deficiency and provides structural insight into a previously undescribed variant. Moreover, early diagnosis and intervention when there is a high index of suspicion may enable the use of less aggressive and less costly treatments.

## Figures and Tables

**Table 1:** Initial Laboratory Values Upon Admission.

Laboratory Parameter (Units)	Normal Range	Case 1	Case 2
Hemoglobin (g/dL)	11.5–13.5	10.7	11.9
White blood cells (1000/µL)	5.00–15.00	7.26	4.87
Platelets (1000/µL)	150–350	718	447
C-Reactive Protein (mg/dL)	0.02–0.5	0.3	0.73
INR	<1.3	1.06	N/A
pH	7.35–7.45	7.37	7.31
pCO_2_ (mmHg)	40–45	23	29.7
**HCO_3_^−^ (calculated HCO_3_^−^) (mmol/L)**	**22.0–26.0**	**13.3 (12.8)**	**17.0 (14.5)**
Serum Sodium [Na] (mEq/L)	135–145	142	139
**Serum Chloride [Cl] (mEq/L)**	**98–106**	**105**	**106**
**Serum Glucose (mg/dL)**	**70–100**	**95**	**128**
**Anion Gap (mEq/L)**	**<14**	**23.7**	**16.0**
Blood Ketones (mmol/L)	0.6–1.5	2.2	N/A
Urinary Ketones	-	+4	N/A
**Aspartate Aminotransferase [AST] (GOT) (U/L)**	**0–31**	**101**	**129**
**Alanine Transaminase [ALT] (GPT) (U/L)**	**0–34**	**370**	**222**
Alkaline Phosphatase [ALP] (U/L)	108–317	238	249
**Gamma-Glutamyl Transferase [GGT] (U/L)**	**4–22**	**31**	17
Albumin (g/dL)	2.8–4.9	4.5	**3.5**
**Lactate (mmol/L)**	**<2.5**	**6.45**	1.90
**Ammonia (µg/dL)**	**18.7–86.9**	**689.8**	**199.0**
Cerebral Spinal Fluid Cell Count (cells/mm^3^)	<5	4	N/A
Cerebral Spinal Fluid **Lactate (mmol/L)**	**1.1–2.8**	**5.4**	N/A

**Table 2:** Laboratory Values Upon Discharge.

Laboratory Parameter (Units)	Normal Range	Case 1	Case 2
pH	7.35–7.45	7.49	7.43
PCO_2_ (mmHg)	40–45	31	35
Serum Sodium [Na] (mEq/L)	135–145	138	139
Serum Chloride [Cl] (mEq/L)	98–106	110	108
HCO_3_^−^ (calculated HCO_3_^−^) (mmol/L)	22.0–26.0	23.6 (22.8)	21.0 (22.4)
Anion Gap (mEq/L)	<14	4.4	10.0
Aspartate Aminotransferase [AST] (GOT) (U/L)	0–31	41	55
Alanine Transaminase [ALT] (GPT) (U/L)	0–34	93	92
Alkaline Phosphatase [ALP] (U/L)	108–317	213	355
Gamma-Glutamyl Transferase [GGT] (U/L)	4–22	38	18
Albumin (g/dL)	2.8–4.9	3.7	4.2
Lactate (mmol/L)	<2.5	1.0	1.2
Ammonia (µg/dL)	18.7–86.9	22	50

## Data Availability

The data presented in this study are available on request from the corresponding author due to ethical considerations and patient confidentiality.
